# Laboratory diffracted x-ray blinking to monitor picometer motions of protein molecules and application to crystalline materials

**DOI:** 10.1063/4.0000112

**Published:** 2021-07-08

**Authors:** Tatsuya Arai, Rena Inamasu, Hiroki Yamaguchi, Daisuke Sasaki, Ayana Sato-Tomita, Hiroshi Sekiguchi, Kazuhiro Mio, Sakae Tsuda, Masahiro Kuramochi, Yuji C. Sasaki

**Affiliations:** 1 Graduate School of Frontier Sciences, The University of Tokyo, Kashiwa 277-8561, Japan; 2 AIST-UTokyo Advanced Operando Measurement Technology Open Innovation Laboratory, National Institute of Advanced Industrial Science and Technology, Kashiwa 277-0882, Japan; 3 Technology and Innovation Center, Daikin Industries, Ltd., 1-1 Nishi Hitotsuya, Settsu-shi, Osaka 566-8585, Japan; 4 Division of Biophysics, Department of Physiology, Jichi Medical University, Shimotsuke, Tochigi 329-0498, Japan; 5 Center for Synchrotron Radiation Research, Japan Synchrotron Radiation Research Institute, 1-1-1, Kouto, Sayo cho, Sayo gun, Hyogo 679-5198, Japan; 6 Bioproduction Research Institute, National Institute of Advanced Industrial Science and Technology, Sapporo 062-8517, Japan; 7 Graduate School of Life Sciences, Hokkaido University, Sapporo 060-0810, Japan

## Abstract

In recent years, real-time observations of molecules have been required to understand their behavior and function. To date, we have reported two different time-resolved observation methods: diffracted x-ray tracking and diffracted x-ray blinking (DXB). The former monitors the motion of diffracted spots derived from nanocrystals labeled onto target molecules, and the latter measures the fluctuation of the diffraction intensity that is highly correlated with the target molecular motion. However, these reports use a synchrotron x-ray source because of its high average flux, resulting in a high time resolution. Here, we used a laboratory x-ray source and DXB to measure the internal molecular dynamics of three different systems. The samples studied were bovine serum albumin (BSA) pinned onto a substrate, antifreeze protein (AFP) crystallized as a single crystal, and poly{2-(perfluorooctyl)ethyl acrylate} (PC_8_FA) polymer between polyimide sheets. It was found that not only BSA but also AFP and PC_8_FA molecules move in the systems. In addition, the molecular motion of AFP molecules was observed to increase with decreasing temperature. The rotational diffusion coefficients (D_R_) of BSA, AFP, and PC_8_FA were estimated to be 0.73 pm^2^/s, 0.65 pm^2^/s, and 3.29 pm^2^/s, respectively. Surprisingly, the D_R_ of the PC_8_FA polymer was found to be the highest among the three samples. This is the first report that measures the molecular motion of a single protein crystal and polymer by using DXB with a laboratory x-ray source. This technique can be applied to any kind of crystal and crystalline polymer and provides atomic-order molecular information.

## INTRODUCTION

Dynamic information within a protein molecule is essential for understanding and controlling the function of that molecule. To date, methodologically, the focus has been on single molecule measurement technology using visible light. However, even if super-resolution technology is used, the internal motion of picometer-sized protein molecules is very difficult to detect because such technology uses wavelengths in the visible light region. It is necessary to acquire a measurement technology that can be used by anyone with a higher positioning accuracy than visible single molecule measurement technology at the laboratory level.

The x-ray single molecule tracking method, called diffracted x-ray tracking (DXT),^1–6^ utilizes the diffraction phenomenon associated with gold nanocrystals labeled onto protein molecules for observation, which enables extremely sensitive measurement of internal molecular dynamics. However, DXT requires the use of white x-rays from a large synchrotron radiation facility to track diffracted x-ray spots from perfect gold nanocrystals. Recently, diffracted x-ray blinking (DXB) using monochromatic x-rays, which can measure the intramolecular movement of proteins as a diffusion coefficient, has been proposed,^7^ and its use is currently expanding.^4,8,9^ In addition, the technique used for producing very good-quality gold nanocrystals, which is essential for DXT and DXB, utilizes a very special technique using epitaxial growth on a single crystal in ultrahigh vacuum. Thus, it is practically difficult for researchers to utilize high-sensitivity high-speed dynamics measurements using DXT and DXB.

**FIG. 1.**
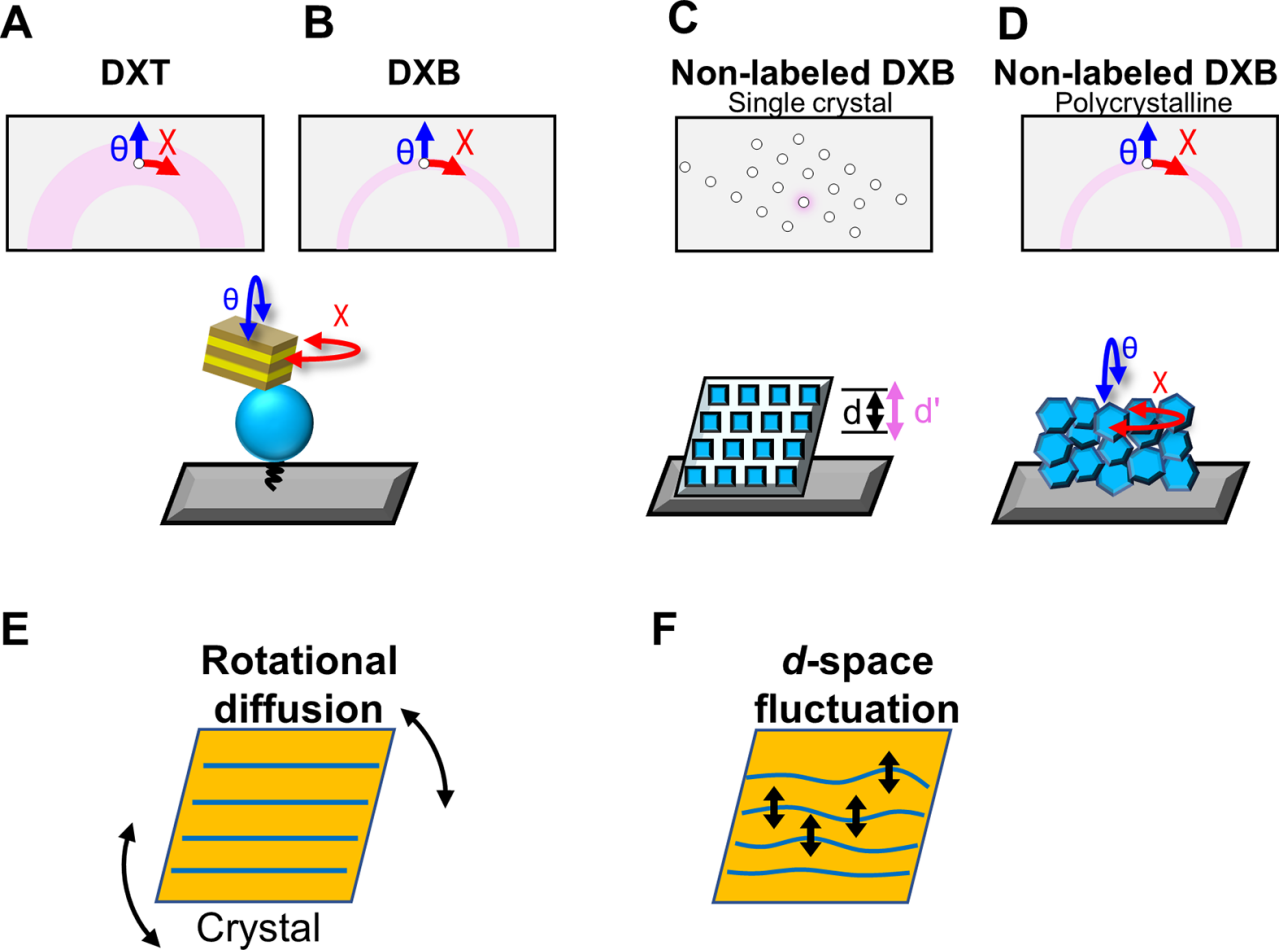
Schematic illustration of DXT (a), DXB (b), and non-labeled DXB using a single crystal (c) and polycrystalline material (d). In (a)–(d), a ring or a half ring colored in pink is the observable region. DXB can measure both rotational diffusion (e) and d-space fluctuation (f).

Figure 1 and Table I compare DXT and DXB with synchrotron and laboratory x-ray sources. Figures 1(a) and 1(b) simply explain the principle of the DXT method using white x-rays from a synchrotron radiation (SR) source and DXB using monochromatic x-rays, respectively. DXT detects the motion of target molecules labeled with a nanocrystal by tracking trajectory of a Laue diffraction spot from the label [Fig. 1(a)]. When monochromatic x-rays are used, movements of diffraction spots behave like on/off blinking at the Debye–Scherrer ring. DXB traces such blinking (intensity fluctuation) that contains information about the molecular motion [Fig. 1(b)]. Note that DXT always needs a SR source, while DXB can theoretically be performed in a laboratory. Since the DXT/DXB measurement targets a noncrystalline system such as single protein molecules, it requires gold nanocrystal labeling. Figures 1(c) and 1(d) show the measurement concept of the DXT/DXB method applied to crystalline samples. If the sample itself is crystalline, it is not necessary to label the gold nanocrystals. By recognizing the measured x-ray diffraction ring as overlapped moving diffraction spots, the motion of fine single crystal particles in the polycrystalline body can be evaluated. Figure 1(c) shows the DXB measurement in the case of a single crystal, while Fig. 1(d) shows the measurement of the sample dynamics in the polycrystalline state. The DXB measurement using a single crystal measures each diffraction spot and is considered to be a time-resolved measurement of the Debye–Waller factor (B factor).^10^ The internal motions of proteins or materials detected by DXT/DXB can be classified into two types, as shown in Figs. 1(e) and 1(f). One is the rotational diffusion motion of a single crystal particle [Fig. 1(e)], such as the motion of labeled gold nanocrystals in DXT shown in Fig. 1(a). The other type is the fluctuation motion of the crystal lattice in the single crystal domain [Fig. 1(f)]. Regarding this motion of the crystal lattice, in addition to the rotational motion, the translational crystal lattice motion also changes the position of the x-ray diffraction spots. Thus, DXT/DXB can detect these summarized motions. The two motions shown in Figs. 1(e) and 1(f) are applied to the DXT/DXB arrangements from Figs. 1(a)–1(d). Since the A and B arrangements use the labeling method, only rotational diffusion motion is detected. Only crystal lattice fluctuation is detected in the Fig. 1(c) arrangement, while both rotational diffusion motion and crystal lattice fluctuation are detected in the Fig. 1(d) arrangement.

**TABLE I.** Comparison of DXT and DXB at laboratory and synchrotron.

	DXT (A)^a^	DXB (B)	DXB (C)	DXB (D)
Target	Single crystal	Single crystal	Single crystal	Polycrystalline
Labeling	Yes	Yes	No	No
X-ray source	SR	Lab/SR	Lab/SR	Lab/SR
X-ray and energy resolution	White ΔE/E ≈ 10^–4^	Monochromatic ΔE/E ≈ 10^–4^–10^–2^	Monochromatic ΔE/E ≈ 10^–4^–10^–2^	Monochromatic ΔE/E ≈ 10^–4^–10^–2^
Analysis concept	Tracking diffraction spots from label	Tracing fluctuation of diffraction intensity from label	Tracing fluctuation of diffraction intensity from target	Tracing fluctuation of diffraction intensity from target
Time resolution	*μ*s	*μ*s (SR)-ms (Lab)	*μ*s (SR)-ms (Lab)	*μ*s (SR)-ms (Lab)

^a^ (A)–(D) correspond to those in Fig. 1.

Here, we applied the DXB measurement to three different samples with laboratory x-ray sources. The first example of our laboratory-DXB is bovine serum albumin (BSA) labeled with commercially available gold colloids. This measurement shows that DXB using commercially available colloidal gold with a laboratory x-ray light source is possible to monitor the protein motion. The second example of the DXB measurement is a single crystal of antifreeze protein (AFP). The final DXB measurement example is a polymer, and its dynamic information can be obtained. However, laboratory x-ray sources cannot provide sufficient flux, so time-resolved x-ray diffraction image data must be carefully statistically processed to extract useful dynamic information.

## MATERIALS AND METHODS

### Sample preparation

An 80 nm gold colloid solution was purchased from BBI Solutions (Cardiff, UK). Crystallized BSA powder was purchased from FUJIFILM Wako Pure Chemical Corporation (Osaka, Japan).

AFP from notched-fin eelpout (nfeAFP) was expressed, purified, and crystallized as previously described.^11^ Briefly, nfeAFP was expressed in *E. coli* and purified by cation exchange chromatography. Purified nfeAFP was crystallized with the hanging-drop vapor-diffusion method. Then, 2–4 *μ*l of 100 mg/ml nfeAFP solution was mixed with 1 *μ*l of crystallization solution [2.5 M ammonium sulfate + 0.1 M sodium citrate buffer (pH 5.0)] and placed onto a polyimide film in a 24-well crystallization plate. After 3–5 days, several 0.5 mm protein crystals were observed and used for the DXB measurement.

Poly{2-(perfluorooctyl)ethyl acrylate} (PC_8_FA) was obtained by free radical polymerization of perfluorooctylethyl acrylate (C_8_FA, Tokyo Chemical Industry Co., Ltd, Tokyo, Japan). More precisely, C_8_FA (10.1 g, 20 mmol) was introduced into a glass tube for the reaction, and then 0.04 g (0.2 mmol) of 2,2′-azobis(isobutyronitrile) (AIBN, FUJIFILM Wako Pure Chemical Corporation) as an initiator was added and reacted at 65 °C with stirring under N_2_ conditions. The reaction was stopped 18 h after AIBN was added. The reacted solution was poured into methanol, and the supernatant was removed to give the remaining white solid. The obtained white solid was dried under vacuum at room temperature for 24 h and then characterized by proton nuclear magnetic resonance (^1^H-NMR) and gel permeation chromatography (GPC). After the white solid was dissolved in a mixed solvent of deuterated chloroform (FUJIFILM Wako Pure Chemical Corporation) and hexafluorobenzene (Tokyo Chemical Industry Co., Ltd), ^1^H-NMR was measured for the white solid by using a JNM-ECZ400S (JEOL Ltd, Tokyo, Japan). The three spectra for the low magnetic field derived from the protons of the acryloyl group of the C_8_FA monomer disappeared, and the spectra derived from the protons of the ethyl group between the fluoroalkyl and ester groups became wider, suggesting that polymerization occurred and PC_8_FA was produced as a white powder. The number average molecular weight (*M*_n_), weight average molecular weight (*M*_w_), and molecular weight dispersity (MWD) of PC_8_FA were determined by GPC measured at DJK Corporation (Kanagawa, Japan). The obtained *M*_n_, *M*_w_, and MWD were 31 000 g/mol, 570 000 g/mol, and 19, respectively.

### Diffracted X-ray blinking

DXB measurements were conducted using a laboratory x-ray source (MicroMax-007 HF: Cu anode, wavelength (λ) = 1.54 Å, 40 kV, 30 mA), and time-resolved diffraction images were recorded using a 2D photon-counting detector (Pilatus 200K-A, Dectris, Switzerland) as shown in Figs. S1(a) and S1(b). The sample-to-detector distances were 30 mm, 60 mm, and 71 mm for BSA, nfeAFP, and PC_8_FA, respectively. The exposure time per frame and interval time were 42.0 ms and 50.0 ms, respectively.

### Single-pixel Autocorrelation Function for DXB analysis

**FIG. 2.**
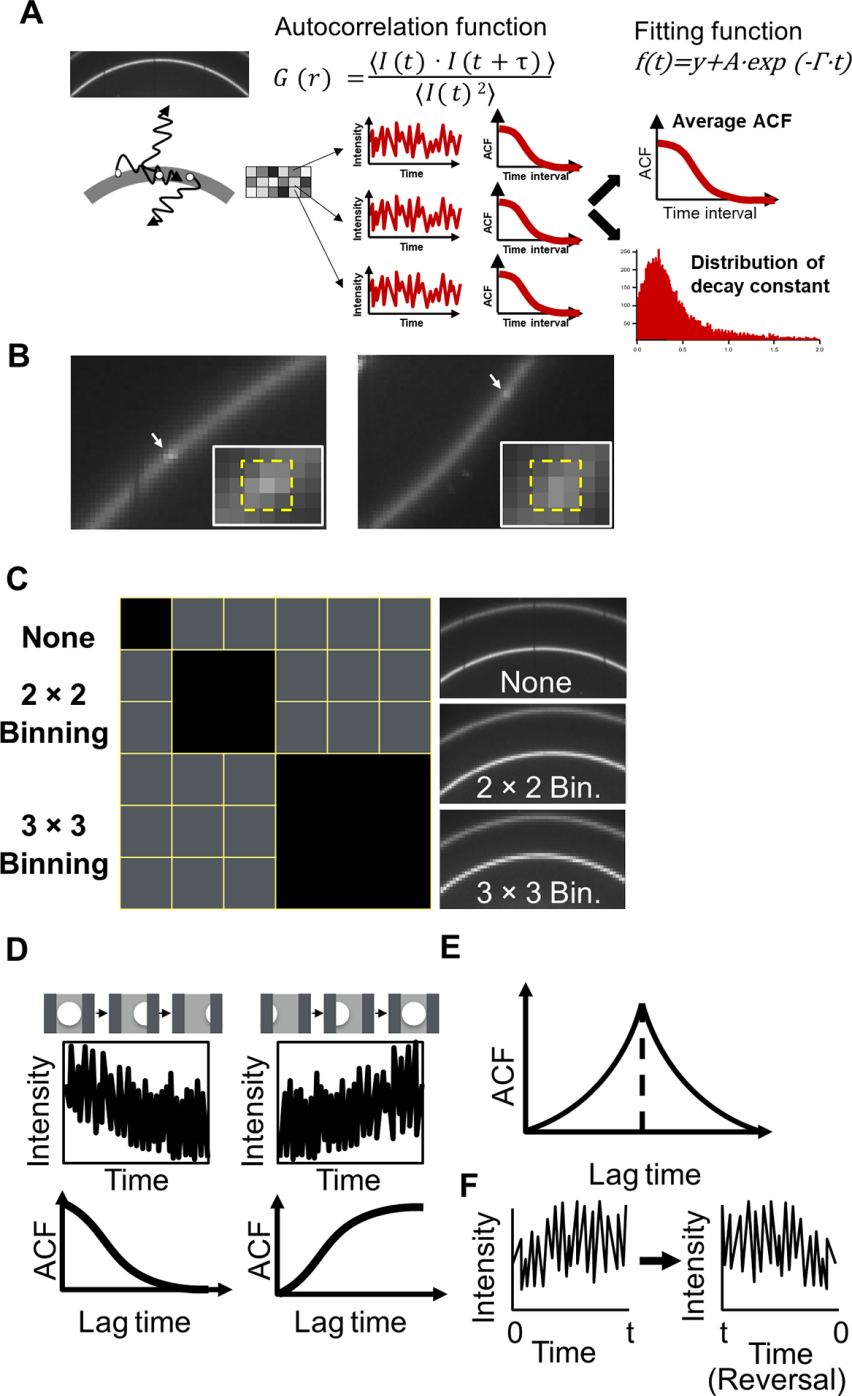
(a) Concept of DXB analysis by using the autocorrelation function (ACF). The panel in the upper left shows an actual XRD image. Time autocorrelation is computed from the intensity fluctuations. (b) The observed diffraction spots on the Debye–Scherrer ring. The size of these spots was approximately 3 × 3 pixels. (c) Schematic illustration of pixel binning. The panel on the right shows actual XRD images after the binning process. (d) Schematic illustration of intensity fluctuation and the ACF used in this study. The diffraction intensity is decreased when the diffraction spots disappear from the Debye–Scherrer ring. ACF exponentially decreases with lag time. On the other hand, the diffraction intensity is increased when the diffraction spots appear on the Debye–Scherrer ring. ACF exponentially increases with lag time. (e) Symmetric property of ACF. (f) Time reversal processing.

The diffraction intensity in each pixel, without the intermodular rectangular area of the detector, was extracted by ImageJ (https://imagej.nih.gov/ij/). When the time-resolved intensity fluctuations showed a long-term trend, such trends were removed by a fitted linear function. The time-resolved intensity trajectory for each pixel was computed by using the following ACF:^4,7,8^

$$I\left(\tau \right)=\frac{\left\langle I\left(t\right)I\left(t+\tau \right)\right\rangle }{\left\langle \;{I\left(t\right)}^{2}\;\right\rangle },$$ (1)

where I(t) represents the diffraction intensity. The brackets ⟨⟩ indicate time averaged values. The computed ACF was fitted to a single exponential curve by ACF(t) = A · exp(−Гt) + y, where A is the amplitude, y is the conversional value, Г is the decay constant, and t is the time interval. The parameters A and y were determined from the computed ACF. The decay constant Г was optimized to fit the ACF curve using a nonlinear least squares method. We chose decay constants to satisfy the following conditions: (I) 0 < y, 0 < A and 0 < Г, and (II) residual values between the fitted and actual ACF curves of less than 1.0. These calculations were performed for all pixels. The averaged ACF and distributions for the decay constants were generated from selected pixels using the above conditions. The distributions of the decay constants were fitted by a Cauchy–Lorentz distribution and were statistically analyzed with the nonparametric Wilcoxon-rank-sum test [Fig. 2(a)].

The rotational diffusion coefficient (D_R_) was calculated by using the following equation as previously described:^8^

$${\;D}_{R}=\frac{\Gamma {{\;\cdot \;\Phi }_{\theta }}^{2}}{4},\;$$ (2)

where Γ is the median value of the ACF decay constant and Φ_θ_ is the rotational displacement.

### Pixel Binning

Pixel binning improves the signal-to-noise ratio (SNR) at the cost of spatial resolution. Diffraction intensities from nanocrystals with an exposure time of 50 ms were low at the laboratory x-ray source. Image processing increases the signal intensity and is probably effective for our ACF analysis. To select a small enough bin size without compromising the quality of the ACF, we carefully examined the diffraction spots on a Debye–Scherrer ring. Few diffraction spots with circular shapes were observed in the laboratory measurements due to the crystalline sample and the incident beam intensity. The size of the observed diffraction spots for gold nanocrystals was approximately 3 × 3 pixels [Fig. 2(b)]. We performed 2 × 2 and 3 × 3 pixel binning for all XRD images. The diffraction intensity is increased in proportion to the number of pixels binned [Fig. 2(c)]. The intensity fluctuation for single pixels after binning probably reflects the motion of single spots. Pixel binning for DXB optimizes the pixel size for diffraction spots and can be used to exactly evaluate the rotational motion of single molecules. In addition, there is also a benefit for carrying out high-speed measurements in laboratory experiments, such as a 50 ms resolution, i.e., the attainment of an improved SNR.

### Time reversal ACF

The ACF decay constant in this paper reflects the mean time in which the diffraction spots disappear from the Debye–Scherrer ring. Actually, ACFs in approximately half of all pixels decayed exponentially. On the other hand, ACFs in the other half gradually increased with lag time [Fig. 2(d)]. The increase in diffraction intensity relates to the entry process of diffraction spots to the Debye–Scherrer ring, which leads to an increase in the ACFs. The autocorrelation function is mathematically an even function^12,13^ [Fig. 2(e)]. Thus, the exit process can be analyzed by using the ACF decay constant after time reversal processing [Fig. 2(f)]. To evaluate the exit process of diffraction spots as the ACF decay constant, we computed ACFs after time reversal processing in each pixel. The ACF decay constant after time reversal processing represents the multiplicative inverse of the mean time in which the diffraction spots enter the Debye–Scherrer ring.

## RESULTS AND DISCUSSION

The reason for choosing the three sample systems for the laboratory DXB measurement is to measure the two different motion patterns [Figs. 1(e) and 1(f)]. The BSA measurement using the labeling method corresponds to Fig. 1(b), and its motion can be identified as rotational diffusion motion. The next example, a protein single crystal, corresponds to Fig. 1(c) and only the fluctuating motion of the crystal lattice can be detected. The final example is a polycrystalline polymer and corresponds to Fig. 1(d). The detected internal motion is assigned to both the rotational diffusion motion of the single crystal domain and the variation dynamics between the crystal lattices within the single crystal domain.

**FIG. 3.**
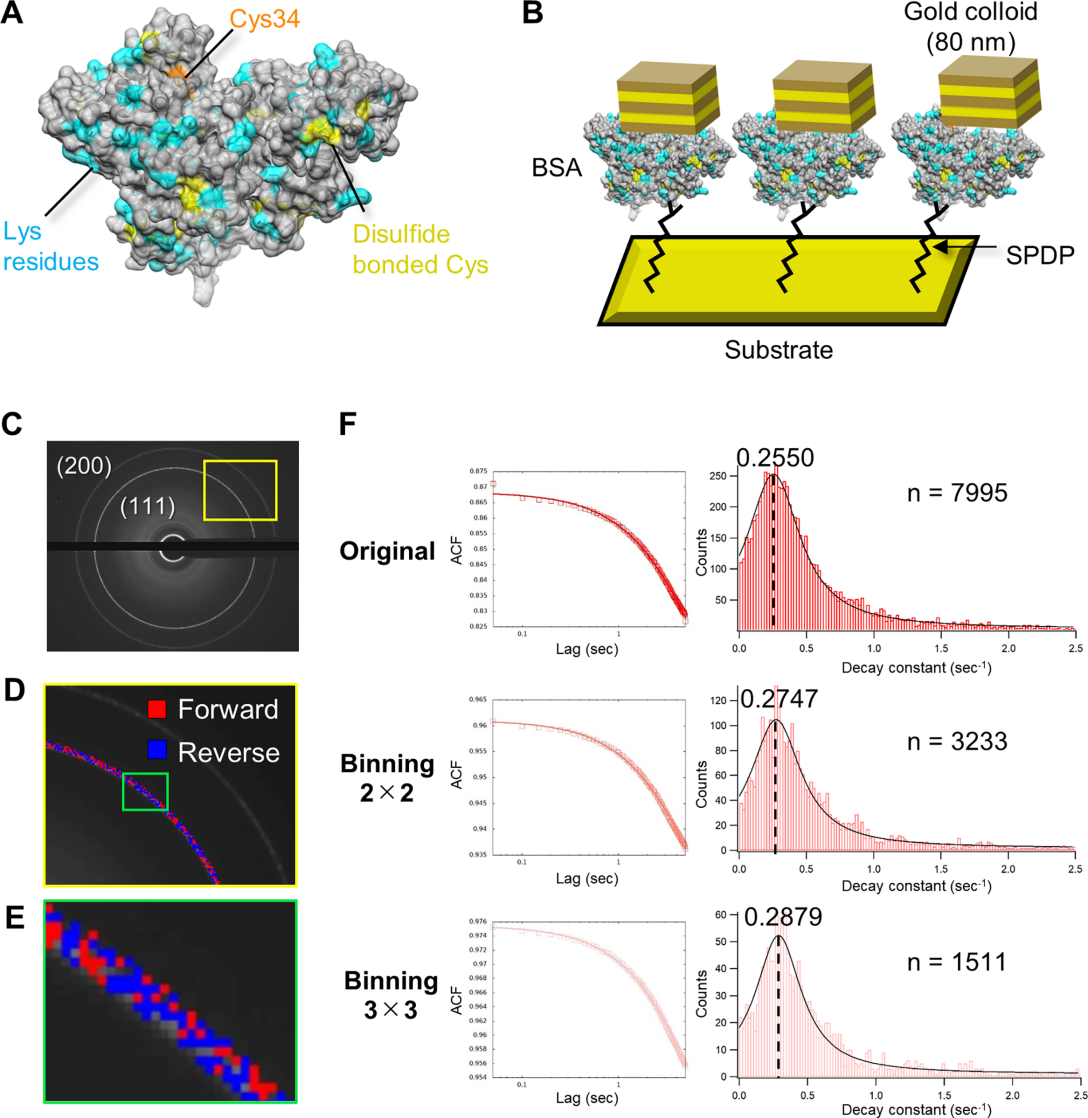
(a) X-ray crystal structure of BSA. (PDB ID: 3V03) shown as a surface model colored cyan, yellow, and orange for lysine, reduced cysteine, and disulfide-bonded cysteine residues, respectively. (b) Schematic illustration of the experimental conditions for DXB. BSA molecules were pinned onto a gold substrate via SPDP. (c) X-ray diffraction pattern for gold nanocolloids. (d) Enlarged image of the yellow box in (c). Red and blue boxes indicate analyzed pixels in which red is analyzed with a normal time course, and blue is analyzed with a reverse time course. (e) Enlarged image of the green box in (d). (f) Average ACF curves and their decay constant histograms for BSA with and without pixel binning. The histograms were fitted with the Cauchy–Lorentz distribution, and the corresponding mode value is shown.

BSA was chosen for the first sample of DXB with a laboratory x-ray source because this 66 kDa globular protein is widely used for a wide variety of experiments as a control. The BSA molecules were labeled with gold nanocrystals through the -SH group of a cysteine residue on its surface [Fig. 3(a)], which is possibly Cys34 because only this residue is in a reduced state for the BSA monomer.^14^ Then, colloidal gold-labeled BSA, as shown in Fig. 3(b), was pinned onto a gold substrate via N-succinimidyl 3-(2-pyridyldithio)propionate (SPDP, Dojindo Laboratories, Kumamoto, Japan), which is a crosslinker between amine (lysine residues of BSA) and sulfhydryl groups (gold substrate). Since the binding site of the gold substrate to the Lys residues for BSA is random, it was assumed that the protein dynamics for BSA in this experiment involved multiple motions. The sample was exposed to x-rays at 20 °C [Fig. S1(c)] (Ref. 28), and time-resolved diffraction images were recorded at a rate of 50 ms/frame for 100 s (2000 frames). X-ray diffraction from the (111) plane of labeled colloidal gold [Fig. 3(c)] was analyzed by using ACF analysis. Since commercially available gold colloids are considerably low crystalline, we were surprised to find that clear diffraction images were obtained from 80 nm diameter gold colloids with laboratory x-ray source tubes. Figures 3(d) and 3(e) show pixels analyzed with the time forward (red) and reversal (blue) ACF analyses, respectively, as described in Figs. 2(d) and 2(e). Similar results were obtained regardless of time direction of ACF analyses (Fig. S2) (Ref. 28). Each pixel was analyzed with either the time-forward or reversal ACF analysis and was not overlapped.

The average ACF curve for BSA and its decay constant distribution are shown in Fig. 3(f) on the left and right, respectively. The adsorbed BSA on the gold substrate undergoes a general Brownian motion in an aqueous solution, and the magnitude of that motion is evaluated by the motion of the colloidal gold. However, the size of the motion cannot be specified in advance, so it is necessary to evaluate what pixel size should be observed. If the optimal motion size of the colloidal gold matches the size of the single-unit pixel obtained by binning (1 × 1, 2 × 2, or 3 × 3), the obtained ACF decay constant will be maximized. The ACF decay constant for BSA increased with increasing binning size, and the maximum decay constant value was obtained when 3 × 3 binning was applied [Fig 3(f) and Table S1]. Thus, the movement of the adsorbed BSA was found to match the pixel size evaluated by 3 × 3 binning. Because the number of statistical data points was dramatically decreased when a binning size of 4 × 4 or more was used, we used a maximum binning size of 3 × 3. The rotational diffusion coefficient (D_R_) was estimated from the ACF decay constant value obtained for 3 × 3 binning and found to have a value of 0.73 pm^2^/s (Table II). These results suggest that millisecond-order protein motion can be measured by using DXB with a laboratory x-ray source.

**TABLE II.** Summary of the results obtained from the ACF analyses.

	Motion	Label	Binning	2θ (°)	*d*-space(Å)	Φ_θ_(pm)	Γ (s^−1^)	D_R_ (pm^2^/s)
BSA	RD^a^	Gold colloid-label	3 × 3	38.24	2.32	3.18	0.29 ± 0.01	0.73 ± 0.01
AFP	DF^b^	Non-label (single)	1 × 1	12.59	7.02	2.55	0.40 ± 0.08	0.65 ± 0.14
PC_8_FA	RD & DF	Non-label (poly)	2 × 2	18.51	4.79	6.51	0.31 ± 0.01	3.29 ± 0.05

^a^ RD: Rotational diffusion.

^b^ DF: *D*-space fluctuation.

**FIG. 4.**
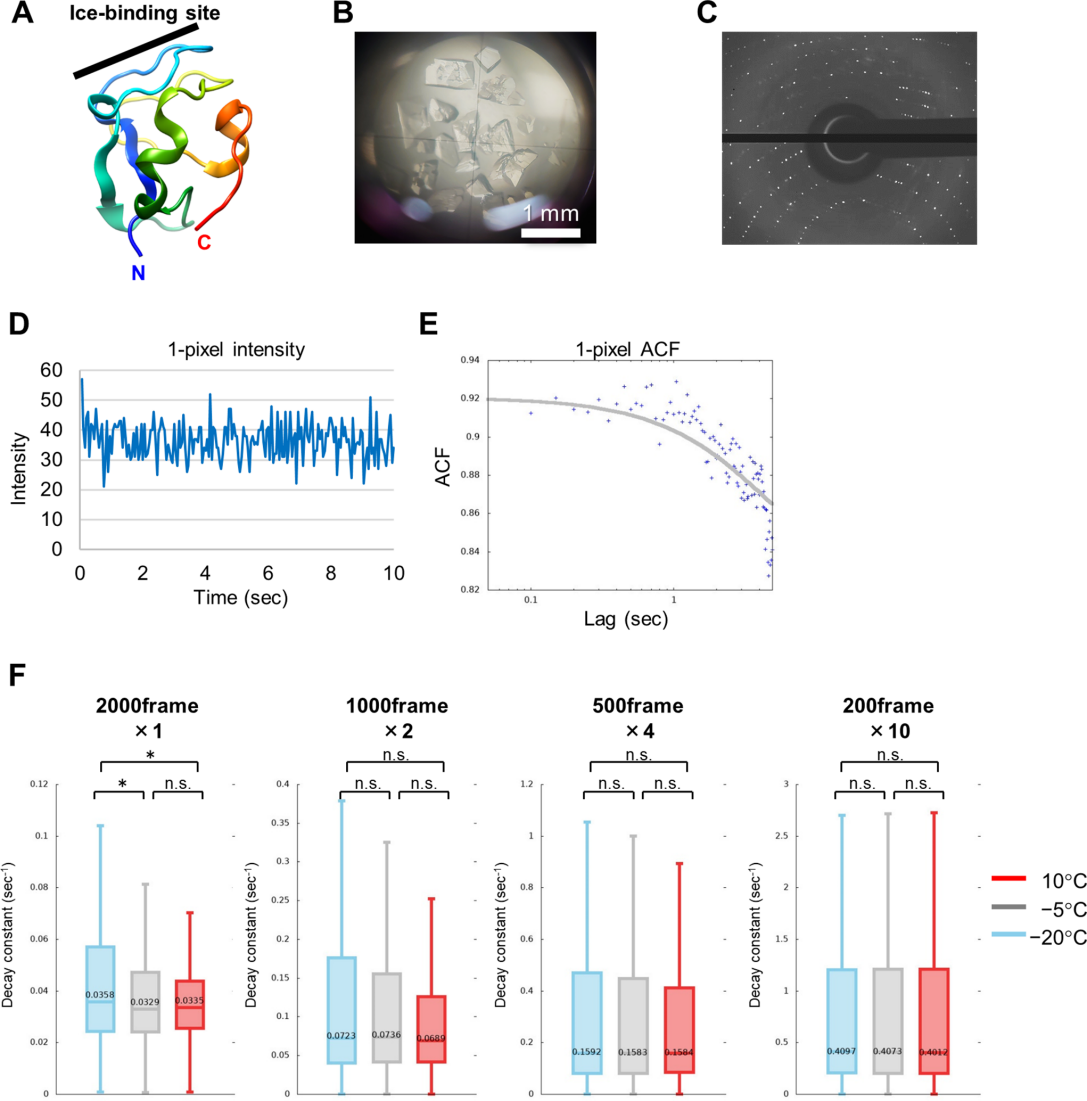
(a) X-ray crystal structure of nfeAFP (PDB: 5XQP) shown as a ribbon model with rainbow coloring from the N-terminus (blue) to the C-terminus (red). (b) nfeAFP single crystals used for non-labeled DXB. (c) X-ray diffraction pattern for a nfeAFP single crystal. (d) Time course of typical x-ray diffraction intensity of a pixel and (e) ACF curve calculated from the pixel. The raw ACF data are shown as blue dots, and its fitting curve is shown in gray and its parameters are ACF(t) = 0.841 + 0.080 · exp(−0.243 · t), where the decay constant Γ is 0.243. (f) Boxplots for the ACF decay constants of the nfeAFP crystal. Median values of the ACF decay constants are shown. ^*^p < 0.05.

Next, DXB was applied to another biomolecule, antifreeze protein (AFP). AFP is a cryoprotective molecule that has the ability to bind to ice crystals to inhibit its growth.^15^ We used a single crystal of AFP to measure non-labeled DXB. Since AFP acts at subzero temperatures, we compared the molecular motion of AFP among different temperatures, which will provide information about the molecular mechanism of AFP. An AFP from notched-fin eelpout^16^ was chosen for this experiment [Fig. 4(a)] because this AFP can be easily crystallized, and its size is relatively large [Fig. 4(b)].^11^ The x-ray crystal and NMR^17^ structure of nfeAFP has been well studied, while the molecular motion of this protein in the crystallized state has never been reported before. nfeAFP was crystallized on a polyimide film in a well of a 24-well crystallization plate. The sample for DXB was prepared by removing the crystallization solution around the AFP crystals and sealing with another polyimide film to avoid drying. The prepared crystals sandwiched with two polyimide films were set on a sample holder. A nfeAFP single crystal in the sample holder was irradiated by laboratory x-rays, and its time-resolved diffraction was collected at a rate of 50 ms/frame. The sample temperature was controlled with a Linkam 10084L temperature-control stage (Linkam Scientific Instruments, UK), and a series of measurements was performed using the same protein crystal without changing the position. Figure 4(c) shows the x-ray diffraction for the nfeAFP crystal. The ACF decay constant was calculated from the intensity fluctuation of each pixel [Figs. 4(d) and 4(e)]. In this case, intensity fluctuations of diffraction spots indicate a slight change in *d*-space, which is possibly ascribed to atomic vibrations in the protein crystal.

In x-ray diffraction experiments of protein single crystals using the synchrotron radiation (SR) source, damage from the x-ray is avoided by measuring it under liquid nitrogen temperature. However, the photon flux of a laboratory x-ray source is as low as 10^8^ photon/s, so that DXB measurement for protein single crystals under the room temperature becomes possible. In fact, there was no change in x-ray diffraction intensity of the sample crystal before and after DXB measurements, while the total irradiation time of our DXB measurement at three difference temperatures was approximately 300 s.

Figure 4(f) shows a comparison of the ACF decay constants among different temperatures. Since the number of pixels was insufficient for fitting to the histogram, we compared the ACF decay constants with boxplots, not histograms. Interestingly, the decay constant of the AFP crystal increased with decreasing temperature, which is inconsistent with Brownian motion. This result was reproducibly observed. A similar trend was reported for another antifreeze protein derived from longsnout poacher (*Brachyopsis segaliensis*).^18^ In this report, the molecular motionality of AFP was determined by a single molecular motion measurement, DXT, and it was observed to increase with decreasing temperature. In addition, it was demonstrated that water solubility of many known species of AFP including nfeAFP is increased dramatically with decreasing the temperature.^19^ Therefore, increasing the molecular vibrating motion of AFP at low temperature might be a common feature among different AFPs and be important for ice binding.

Two thousand frames of 50 ms x-ray diffraction images were divided into two, four, and ten data sets of 1000, 500, and 200 frames, respectively, and these three data sets were again analyzed with ACF [Fig. 4(f)]. As the number of frames decreased, the differences in the decay constants among temperatures became ambiguous. Decreasing the number of frames means shortening the total observation time. Therefore, long-term observation is required to detect the characteristic motion in AFP crystals.

**FIG. 5.**
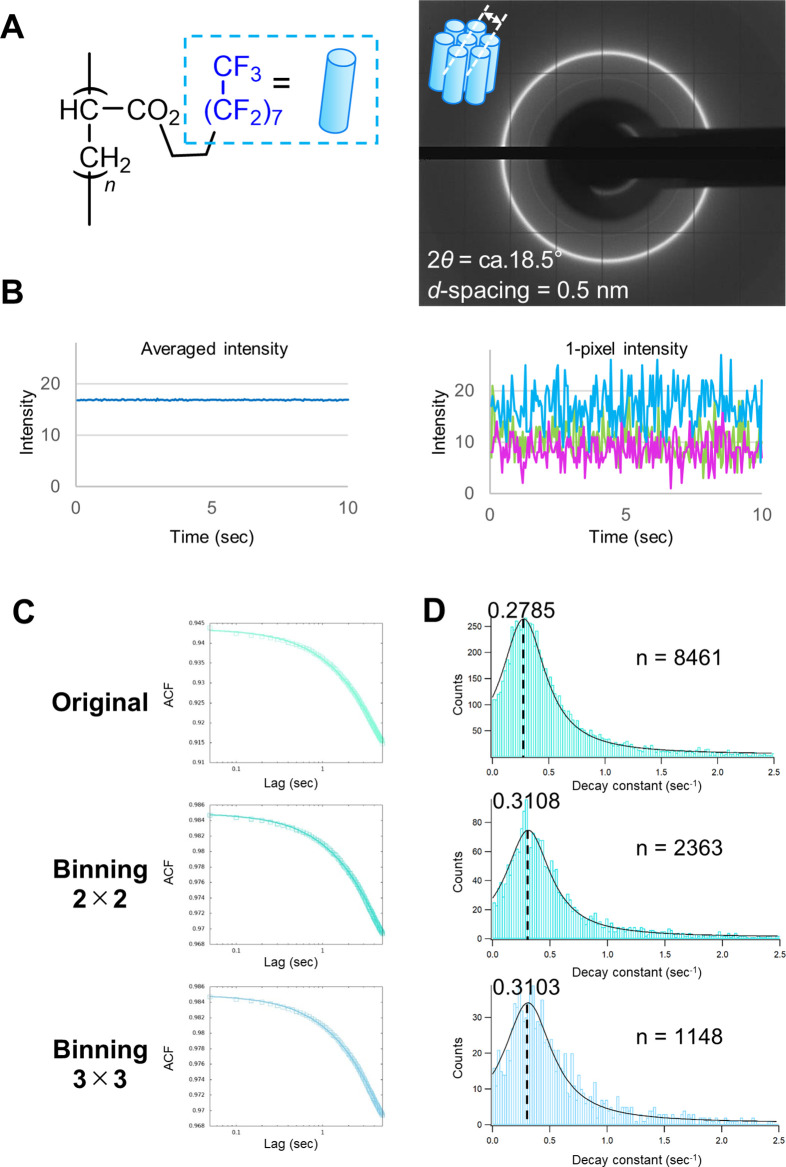
(a) Chemical structure of PC_8_FA. Schematic illustration of the fluoroalkyl groups at the side chain of the polymer and the x-ray diffraction pattern attributed to the fluoroalkyl groups at the side chain of PC_8_FA. (b) Time-trajectories of averaged x-ray diffraction intensity (left) and typical three pixel (right). (c) ACF curves calculated from the diffraction intensity fluctuations of PC_8_FA. (d) Decay constant histograms for the ACF decay constant for PC_8_FA. The histograms were fitted with a Cauchy–Lorentz distribution, and the corresponding mode value is shown.

Finally, DXB was applied for molecular dynamics measurement on a polymer with water/oil repellent performance, PC_8_FA. This polymer has fluoroalkyl groups at the side chains and forms a hexagonal packing.^20–22^ The detailed dynamic observation of molecular state of fluoroalkyl groups at the side chain of PC_8_FA on a very short timescale has never been measured directly before, although it was previously suggested that its water repellent ability is related to crystal packing (rigidity) of the fluoroalkyl groups.^21^ However, the mechanism of many polymer physical properties is often still unknown. Since the movement of a polymer chain contributes to its performance, it is suggested that if non-labeled DXB, which is easily measured by using laboratory x-ray equipment, can observe the movement of a polymer chain, it is very useful and valuable both academically and industrially. Therefore, we measured the molecular dynamics of PC_8_FA as a model for measuring polymer dynamics using non-labeled DXB. The test piece used for DXB measurement was prepared by placing PC_8_FA onto a polyimide sheet, heating it to 130 °C to melt it, and then sandwiching it with another polyimide sheet. Figure 5(a) shows that the x-ray diffraction pattern for PC_8_FA was observed at 2*θ* = *ca.* 18.5°, and its *d*-space was estimated to be 4.8 Å.^20–24^ Figure 5(b) left side is the averaged intensity of the analyzed x-ray diffraction, and Fig. 5(b) right side shows diffraction intensities of typical three pixels. These time-trajectories of diffraction intensities were used for ACF analysis, and averaged ACF curves are shown in Fig. 5(c). Figure 5(d) shows histograms for the calculated ACF decay constants, and their median values were estimated by Cauchy–Lorentz fitting (Table S3). The rotational diffusion coefficient of PC_8_FA was determined to be 0.81 pm^2^/s. These results suggest that the fluoroalkyl groups at the side chain of PC_8_FA are moving or vibrating in a very densely aggregated state generally called a crystalline state at the picometer scale. The ACF decay constant of PC_8_FA decreased with increasing binning size from 1 × 1 to 2 × 2, while it did not change when increasing the binning size from 2 × 2 to 3 × 3. This result shows that the size of the molecular internal dynamics for PC_8_FA roughly matches the pixel size of 2 × 2. The rotational diffusion coefficient (D_R_) of PC_8_FA with 2 × 2 binning was estimated to be 3.29 pm^2^/s (Table II). The D_R_ value for PC_8_FA is larger than that for BSA (0.73 pm^2^/s). The reason for this seemingly contradictory behavior might be that BSA molecules could be pinned on the substrate via multiple lysine residues [Figs. 3(a) and 3(b)], which physically suppresses its rotational motion. Thus, it is necessary to well consider the method for fixing the molecules to the substrate, which may affect the molecular motion.

In the current study, we measured the molecular motion of different kinds of samples at a laboratory. Such molecular motion can be measured by super-resolution microscopy (SRM)^25,26^ and fluorescence resonance energy transfer (FRET) spectroscopy.^27^ Maximum resolution of these techniques is nanometer-order. By contrast, DXB can monitor the picometer-order of molecular motion. In addition, SRM and FRET can measure neither semi-crystalline polymer nor protein in the crystal. Thus, it can be said that laboratory DXB is a prospective technique and will provide novel information to understand molecular behavior.

## CONCLUSION

Using the laboratory DXB method, we succeeded in measuring the internal dynamics of molecules in three different systems. This is the first report in which a laboratory x-ray source was utilized to evaluate the dynamic characteristics of crystalline materials. Due to the low x-ray intensity and time resolution limit of the x-ray detector used, the measured time axis covered a 50 ms region; however, picometer-sized internal molecular motion was detected.
